# Household sanitation facilities and women’s risk of non-partner sexual violence in India

**DOI:** 10.1186/s12889-016-3797-z

**Published:** 2016-11-08

**Authors:** Apoorva Jadhav, Abigail Weitzman, Emily Smith-Greenaway

**Affiliations:** 1 University of Michigan, 426 Thompson St, Ann Arbor, MI 48104 USA; 2 University of Southern California, 851 Downey Way, Los Angeles, CA 90089 USA

**Keywords:** Non-partner sexual violence, Rape, Sanitation, Defecation, Child health

## Abstract

**Background:**

Globally, one in ten individuals practice open defecation. Despite media speculation that it increases women’s risk of sexual violence, little empirical evidence supports the claims. We investigate the relationship between household sanitation facilities and women’s risk of non-partner sexual violence (NPSV) in India, where nearly half of the population lives without a pit or toilet.

**Methods:**

We use the most recent NPSV data, from the National Family Health Survey-III, to estimate logistic regression models of the effects of household sanitation facilities (toilet, pit, or none) on NPSV in the last year among women who have resided in their current home for one year or more. These effects are estimated net of other socioeconomic factors, compared to effects of household sanitation facilities on child diarrhea, and, as a falsification test, compared to effects of household sanitation facilities on intimate partner sexual violence (IPSV) in the last year.

**Results:**

Net of their socioeconomic status, women who use open defecation are twice as likely to face NPSV as women with a household toilet. This is twice the association between open defecation and child diarrhea. The results of our falsification test indicate that open defecation is not correlated with IPSV, thus disconfirming a simultaneous selection of women into open defecation and sexual violence.

**Conclusions:**

Our findings provide empirical evidence that lacking household sanitation is associated with higher risk of NPSV.

## Background

Nearly half of the world’s population (42 %) continues to lack access to improved sanitation conditions, with more than one in ten (13 %) individuals forced to defecate in the open [1]. The majority of these individuals (59 %) reside in India. Since 1990, the Indian government has introduced nationwide campaigns to improve sanitation facilities across the country—first under the Total Sanitation Campaign (TSC), and most recently under the Swachh Bharat Mission (SBM) [2]. However, the lack of improved sanitation remains a major public health concern [1, 3].

To date, public health research on open defecation has centered on its link to multiple infectious diseases, with a focus on its connection to ill health, particularly among children, who are especially vulnerable to diarrhea-related morbidity [4, 5]. However, more recently, media has highlighted how poor sanitation extends beyond specific disease etiologies, suggesting that open defecation may put women at higher risk of NPSV [6]. Despite media accounts suggesting this link, scant empirical evidence exists., Qualitative research has linked women’s lack of household sanitation and clean water sources to a heightened fear of sexual violence in India [7, 8], Vietnam [8], Kenya [9], and Ghana [10]. In the Indian state of Orissa, a majority of respondents feared unwanted sexual encounters such as being watched, indecent exposure, and NPSV. Another study confirmed that adolescent girls and young women—especially those residing in the local slum—were sexually victimized while accessing sanitation sources [11]. One recent study of women in Kenya linked open defecation to higher odds of NPSV [12].

We contribute to this body of literature by examining whether women’s lack of household sanitary facilities is associated with a higher likelihood of experiencing NPSV. We use the most recent data available on sexual violence in India to test this relationship, estimating the effects net of other socioeconomic characteristics, and employing information from time-specific questions that ensure household sanitation facilities preceded violent incidents. We further rule out selection bias with a falsification test in which we use household sanitation facilities to predict intimate partner sexual violence (IPSV). To contextualize the magnitude of our findings, we conduct a parallel analysis of the relationship between household sanitation facilities and risk of waterborne illness, specifically, child diarrhea.

Our study is an important extension of research on violence against women, exploring a predictor of NPSV that is not well researched. While the data are from 2005-06, they are the most recently available data on sexual violence in India. Moreover, there is no reason to believe that the effects of open defecation on NPSV are period specific. We examine this association by joining two distinct literatures on sexual violence and water, sanitation and hygiene. It has been pointed out that discussions about making progress toward Millennium Development Goal (MDG) 7c – to “halve, by 2015, the proportion of the population without sustainable access to safe drinking water and basic sanitation” – have overlooked the importance of this goal for women’s safety [13]. There is a critical need to understand the implications of sanitation facilities beyond the realm of illness. We argue that access to sanitation is a major factor in understanding sexual violence against women, and utilizing toilets may substantially mitigate some women’s risk of NPSV.

## Methods

We analyze data from the 2005–06 Indian National Family Health Survey- III (NFHS), a nationally representative dataset collected by ICF international and the Indian Ministry of Health using a stratified random sampling approach [14]. 68 % of women from the full sample were selected for the domestic violence module, with one woman between the ages of 15 and 49 randomly selected from each household to answer questions about their exposure within the past year to different types of violence, including NPSV. Nearly all women agreed to participate in the module (99 %), which was administered in a private setting to help ensure accuracy of reports. We exclude the small percentage (4 %; *N* = 2,908) of women who had not resided in their current household for at least one year at the time of the survey, given that we have data on the sanitary facilities solely in women’s current household. Because the measure of household sanitary facilities is at a single point in time, it is possible they changed over the course of the preceding year. The final analytic sample consists of 75,619 women.

To analyze the relationship between household sanitation facilities and NPSV, we classify women’s household sanitation facilities into three categories: (1) toilet (i.e., flush, compost, and dry toilets), (2) pit/latrine, and (3) open defecation, which refers to respondents whose property does not contain any type of sanitation facility. We distinguish sexual violence from physical violence using World Health Organization (WHO) definitions. They define physical violence – being pushed, kicked, shoved, dragged on purpose – largely in the realm of intimate partner violence (IPV), while non partner violence is defined largely by sexual violence. Specifically, as any woman aged 15 and over being forced to perform any sexual act that they did not want, by someone other than husband or partner [15]. Our measure of NPSV is based on responses to two questions. First, all women were asked: “At any time in your life, as a child or as an adult, has anyone ever forced you in any way to have sexual intercourse or perform any other sexual acts?” Never-married respondents who replied “yes” were then asked: “In the last twelve months, has anyone forced you to have sexual intercourse or perform any other sexual acts against your will?” Ever-married respondents who replied “yes” were asked the same question but were specifically asked whether “anyone other than your (current/last) husband” had forced them to perform such acts. ‘Yes’ answers from both never- and ever-married respondents are coded 1; ‘no’ answers for ever experiencing sexual violence or for experiencing sexual violence from someone other than a husband in the prior twelve months are coded 0. Twelve respondents refused to answer the question and are omitted from the analysis (<0 · 01 % of the sample). No information about the perpetrators, or report of NPSV to authorities, or severity of attack was asked in this survey, which would have added to our understanding of NPSV in India.

We begin our investigation with a bivariate analysis of household sanitation facilities and NPSV. We then conduct a multivariate analysis that adjusts for respondents’ demographic characteristics, including number of years of education (0–23); age (15–49 years); relationship status (never married, married, widowed, not living together); caste and religion (upper caste Hindu; scheduled caste/tribe Hindu; other backward caste Hindu; lower class Muslim; forward class Muslim; other scheduled caste/tribe, Christian, or Buddhist; and other); level of urbanization (mega city, large city, small city, large town, small town, rural); and geographic region (North, North-central, North-east, East, West, and South). Last, we rerun our multivariate model of NPSV adjusting for both demographic characteristics and household assets, which are characterized using both a dummy for electricity and a categorical measure of roof material (natural; man-made and semi-permanent; and man-made and permanent). Because NPSV is measured dichotomously, we use logistic regressions to conduct all bivariate and multivariate analyses.

We estimate two parallel sets of analyses. First, to put the magnitude of our estimated associations of the relationship between women’s access to sanitation facilities and NPSV in perspective, we test the relationship between household sanitation facilities and diarrhea among children. This analysis is limited to mothers with a child younger than age five residing in their same household (*N* = 25,285; 33 % of the analytic sample). Among respondents with children of this age, those who report that at least one child has had diarrhea within the preceding two weeks are coded (1) for “at least one child sick”; those who report that no child has had diarrhea within the preceding two weeks are coded (0) for “none.” Among this sample of respondents, the proportions reporting at least one young child with diarrhea are 10 % (95 % CI 0 · 09-0 · 10) for those using a toilet, 13 % (95 % CI 0 · 11-0 · 14) for those using a pit/latrine, and 12 % (95 % CI 0 · 11-0 · 13) for those using open defecation (bivariate analysis not shown).

Second, to address whether the observed associations with NPSV reflect the selection of disadvantaged women into both households that lack sanitation facilities and higher risk of sexual violence, or are specific only to NPSV, we explore whether lack of sanitation facilities is also associated with IPSV. Though we account for socioeconomic factors in our main analyses, we recognize that other unobserved factors could drive a spurious association between household sanitation facilities and sexual violence. However, if this were the case, we would also anticipate to observe an association between household sanitation facilities and IPSV. Because there is no reason to believe there is a direct association between household sanitation facilities and IPSV, documenting such an association would suggest our findings are spurious. However, finding no direct association between sanitation and IPSV would strengthen support that any observed association between household sanitation facilities and NPSV reflects a non-spurious process. Among the full sample, proportions reporting IPSV in the prior 12 months are 5 % (95 % CI 0 · 04-0 · 05) for those using a toilet and 8 % for those using either a pit/latrine (95 % CI 0 · 07-0 · 09) or open defecation (95 % CI: 0 · 07-0 · 08).

In our analyses of both child diarrhea, and IPSV, we adjust for the same demographic characteristics and household assets as our most saturated model of NPSV. All analyses are weighted to provide nationally representative estimates. For ease of interpretation, we present the results of all logistic regressions in terms of odds-ratios (Tables 2, 3 and 4).

## Results

**Fig. 1:**
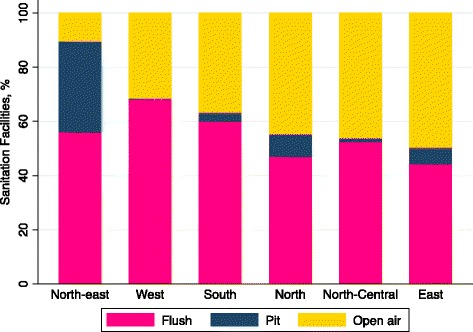
Percentage households with flush toilet, pit latrine, or reliance on open air defecation in India, by region (*N* = 75,619 households)

Only 53 % (95 % CI 0 · 52-0 · 53) of respondents in our sample have access to sanitation facilities in the household; 8 % (95 % CI 0 · 08-0 · 08) use pits/latrines; and 39 % (95 % CI 0 · 38-0 · 39) use open defecation (univariate analysis not shown). As shown in Fig. 1, women’s access to household sanitation facilities varies substantially across regions of India. For instance, in Northeast India, only 10 % (95 % CI 0 · 10-0 · 11) of respondents defecate in the open compared to 50 % (95 % CI 0 · 49-0 · 50) of respondents in East India.

As presented in Table 1, NPSV is rare in the Indian context. According to our estimates, 0 · 1 % (95 % CI 0 · 001-0 · 001) of respondents using toilets, 0 · 1 % (95 % CI 0 · 0004-0 · 002) of respondents using pits/latrines, and 0 · 2 % (95 % CI 0 · 001-0 · 002) of respondents using open defecation had experienced NPSV in the prior year. Despite its rarity, our descriptive analysis indicates that NPSV is twice as common among women using open defecation than it is among women using toilets (t-tests confirm this difference; *p* < 0.05), though we found no statistically significant difference in risk of NPSV among women using pits/latrines versus open defecation (analysis not shown).

**Table 1:** Sample characteristics of Indian Women, by their household’s sanitation facilities (2005-06), *N* = 75,619 women

	Toilet		Pit/ latrine		Open air	
	Mean	(95 % CI)	Mean	(95 % CI)	Mean	(95 % CI)
*Outcomes*						
Non-partner sexual violence within 12 months	0 · 001	(0 · 001 – 0 · 001)	0 · 001	(0 · 0004 – 0 · 002)	0 · 002	(0 · 001 – 0 · 002)
*Respondent’s demographic characteristics*						
Education (years)	7 · 75	(7 · 71 – 7 · 80)	4 · 96	(4 · 85 – 5 · 07)	2 · 45	(2 · 41 – 2 · 49)
Age (years)	30 · 77	(30 · 69 –30 · 86)	29 · 87	(29 · 66 – 30 · 09)	29 · 83	(29 · 73 – 29 · 93)
Relationship status						
Never married	0 · 18	(0 · 17 – 0 · 18)	0 · 15	(0 · 14 – 0 · 16)	0 · 11	(0 · 11 – 0 · 12)
Married	0 · 79	(0 · 78 – 0 · 79)	0 · 81	(0 · 80 – 0 · 82)	0 · 83	(0 · 83 – 0 · 84)
Widowed	0 · 03	(0 · 03 – 0 · 03)	0 · 03	(0 · 02 – 0 · 03)	0 · 04	(0 · 04 – 0 · 04)
Not living together	0 · 01	(0 · 01 – 0 · 01)	0 · 02	(0 · 01 – 0 · 02)	0 · 01	(0 · 01 – 0 · 02)
Caste/ religion						
Upper Caste Hindu	0 · 34	(0 · 34 – 0 · 35)	0 · 25	(0 · 24 – 0 · 26)	0 · 14	(0 · 14 – 0 · 14)
SC/ST Hindu	0 · 14	(0 · 14 – 0 · 14)	0 · 18	(0 · 17 – 0 · 19)	0 · 33	(0 · 33 – 0 · 34)
OBC Hindu	0 · 28	(0 · 27 – 0 · 28)	0 · 19	(0 · 18 – 0 · 20)	0 · 40	(0 · 39 – 0 · 40)
Lower class Muslim	0 · 06	(0 · 06 – 0 · 06)	0 · 04	(0 · 03 – 0 · 04)	0 · 04	(0 · 04 – 0 · 04)
Forward class Muslim	0 · 10	(0 · 09 – 0 · 10	0 · 25	(0 · 24 – 0 · 26)	0 · 05	(0 · 05 – 0 · 06)
Other SC/ST, Christian, Buddhist	0 · 04	(0 · 03 – 0 · 04)	0 · 05	(0 · 05 – 0 · 06)	0 · 02	(0 · 02 – 0 · 02)
Other	0 · 05	(0 · 05 – 0 · 05)	0 · 04	(0 · 04 – 0 · 05)	0 · 02	(0 · 02 – 0 · 02)
City size						
Mega city	0 · 08	(0 · 08 – 0 · 08)	0 · 004	(0 · 002 – 0 · 01)	0 · 001	(0 · 001 – 0 · 002)
Large city	0 · 17	(0 · 17 – 0 · 18)	0 · 05	(0 · 04 – 0 · 05)	0 · 01	(0 · 01 – 0 · 01)
Small city	0 · 18	(0 · 17 – 0 · 18)	0 · 05	(0 · 05 – 0 · 06)	0 · 02	(0 · 02 – 0 · 03)
Large town	0 · 04	(0 · 04 – 0 · 05)	0 · 02	(0 · 02 – 0 · 02)	0 · 01	(0 · 01 – 0 · 01)
Small town	0 · 17	(0 · 17 – 0 · 17)	0 · 11	(0 · 10 – 0 · 12)	0 · 06	(0 · 05 – 0 · 06)
Rural	0 · 35	(0 · 35 – 0 · 36)	0 · 77	(0 · 76 – 0 · 78)	0 · 90	(0 · 90 – 0 · 90)
Geographic region						
North	0 · 11	(0 · 11 – 0 · 11)	0 · 12	(0 · 11 – 0 · 13)	0 · 11	(0 · 10 – 0 · 11)
North– central	0 · 23	(0 · 23 – 0 · 24)	0 · 13	(0 · 12 – 0 · 14)	0 · 35	(0 · 35 – 0 · 36)
North– east	0 · 04	(0 · 04 – 0 · 04)	0 · 32	(0 · 30 – 0 · 33)	0 · 01	(0 · 01 – 0 · 02)
East	0 · 13	(0 · 13 – 0 · 14)	0 · 26	(0 · 25 – 0 · 27)	0 · 19	(0 · 18 – 0 · 19)
West	0 · 21	(0 · 20 – 0 · 21)	0 · 01	(0 · 01 – 0 · 02)	0 · 12	(0 · 12 – 0 · 13)
South	0 · 28	(0 · 27 – 0 · 28)	0 · 16	(0 · 15 – 0 · 17)	0 · 22	(0 · 21 – 0 · 22)
*Household Assets*						
Has electricity in house	0 · 92	(0 · 92 – 0 · 92)	0 · 61	(0 · 60 – 0 · 62)	0 · 52	(0 · 52 – 0 · 53)
Roof material						
Natural	0 · 06	(0 · 06 – 0 · 06)	0 · 20	(0 · 19 – 0 · 21)	0 · 32	(0 · 32 – 0 · 33)
Man– made, semi– permanent	0 · 18	(0 · 18 – 0 · 19)	0 · 40	(0 · 39 – 0 · 41)	0 · 16	(0 · 15 – 0 · 16)
Man– made, permanent	0 · 75	(0 · 75 – 0 · 76)	0 · 40	(0 · 39 – 0 · 41)	0 · 52	(0 · 52 – 0 · 53)

The logistic regression model results shown for Model 1 of Table 2 confirm a significant association between household sanitary facilities and NPSV. Compared to women who have access to a toilet in their household, Indian women who must open defecate have 2.14 times the risk of NPSV (*p* < .01). The results for Model 2 – which includes the demographic, geographic, and socioeconomic factors – confirm the association is robust, though the strength of the association is slighted attenuated (*p* < .05). The results for Model 3 – which adds in measures of household assets and infrastructure – also support the significant association between open defecation and NPSV. Analysis including interaction terms with demographic and socioeconomic correlates was not significant.

**Table 2:** Logistic regression model results of the association between Indian women’s household sanitation facilities and their experience of NPV within the last twelve months (2005-06), *N* = 75,619

	Model 1		Model 2		Model 3 (Full model)	
	Bivariate		Adjusted for demographic characteristics		Adjusted for demographic characteristics and household assets	
	Odds ratio		Odds ratio		Odds ratio	
	(95 % CI)	*p*-value	(95 % CI)	*p*-value	(95 % CI)	*p*-value
Sanitation facilities						
Toilet	1	NA	1	NA	1	NA
Pit	1 · 29	0 · 67	0 · 58	0 · 31	0 · 59	0 · 33
	(0 · 40 – 4 · 19)		(0 · 20 – 1 · 66)		(0 · 20 – 1 · 70)	
Open air	2 · 14	0 · 004	2 · 15	0 · 02	2 · 25	0 · 02
	(1 · 28 – 3 · 56)		(1 · 11 – 4 · 15)		(1 · 13 – 4 · 50)	
*Respondent’s demographic characteristics*						
Education			1	0 · 98	1	0 · 93
			(0 · 93 – 1 · 08)		(0 · 93 – 1 · 07)	
Age			0 · 96	0 · 014	0 · 96	0 · 01
			(0 · 93 – 0 · 99)		(0 · 93 – 0 · 99)	
Relationship status						
Never married			1	NA	1	NA
Married			0 · 52	0 · 09	0 · 52	0 · 09
			(0 · 24 – 1 · 11)		(0 · 24 – 1 · 11)	
Widowed			3 · 54	0 · 03	3 · 52	0 · 03
			(1 · 14 – 10 · 99)		(1 · 13 – 10 · 95)	
Not living together			0 · 69	0 · 67	0 · 70	0 · 68
			(0 · 13 – 3 · 73)		(0 · 13 – 3 · 79)	
Caste/ religion						
Upper caste Hindu			1	NA	1	NA
SC/ST Hindu			2 · 71	0 · 02	2 · 74	0 · 02
			(1 · 17 – 6 · 31)		(1 · 18 – 6 · 34)	
OBC Hindu			1 · 44	0 · 43	1 · 44	0 · 42
			(0 · 59 – 3 · 50)		(0 · 59 – 3 · 50)	
Lower class Muslim			6 · 75	<0 · 001	6 · 75	<0 · 001
			(2 · 36 – 19 · 29)		(2 · 36 – 19 · 32)	
Forward class Muslim			2 · 62	0 · 11	2 · 60	0 · 11
			(0 · 80 – 8 · 59)		(0 · 81 – 8 · 35)	
Other SC/ST, Christian, Buddhist			1 · 62	0 · 42	1 · 64	0 · 41
			(0 · 50 – 5 · 18)		(0 · 51 – 5 · 25)	
Other			0 · 38	0 · 05	0 · 37	0 · 04
			(0 · 15 – 0 · 99)		(0 · 14 – 0 · 97)	
City size						
Mega city			1	NA	1	NA
Large city			9 · 79	0 · 04	9 · 78	0 · 04
			(1 · 10 – 86 · 72)		(1 · 10 – 87 · 33)	
Small city			9 · 34	0 · 05	9 · 27	0 · 05
			(1 · 04 – 84 · 09)		(1 · 03 – 83 · 32)	
Large town			11 · 28	0 · 07	11 · 16	0 · 08
			(0 · 80 – 159 · 48)		(0 · 78 – 160 · 26)	
Small town			7 · 95	0 · 05	7 · 95	0 · 05
			(0 · 97 – 65 · 07)		(0 · 97 – 65 · 40)	
Rural			7 · 46	0 · 06	7 · 55	0 · 06
			(0 · 93 – 59 · 77)		(0 · 95 – 60 · 31)	
Geographic region						
North			1	NA	1	NA
North-central			0 · 92	0 · 85	0 · 90	0 · 82
			(0 · 40 – 2 · 12)		(0 · 39 – 2 · 11)	
North-east			4 · 64	0 · 002	4 · 22	0 · 01
			(1 · 77 – 12 · 20)		(1 · 41 – 12 · 63)	
East			0 · 98	0 · 97	0 · 90	0 · 81
			(0 · 42 – 2 · 30)		(0 · 38 – 2 · 14)	
West			0 · 86	0 · 78	0 · 76	0 · 61
			(0 · 28 – 2 · 58)		(0 · 26 – 2 · 22)	
South			0 · 60	0 · 34	0 · 58	0 · 30
			(0 · 22 – 1 · 69)		(0 · 21 – 1 · 60)	
*Household assets*						
Electricity in home					0 · 95	0 · 86
Roof material					(0 · 51 – 1 · 75)	
Natural					1	NA
Man-made, semi-permanent					1 · 65	0 · 26
					(0 · 69 – 3 · 93)	
Man-made, permanent					1 · 54	0 · 23
					(0 · 77 – 3 · 08)	
Constant	0 · 001	<0 · 001	0 · 0003	<0 · 001	0 · 0002	<0 · 001
	(0 · 001 – 0 · 001)		(0 · 00003 – 0 · 003)		(0 · 00002 – 0 · 003)	

*Note. 95 % CI in parentheses*

To put the magnitude of this association in perspective, Table 3 presents the results of a parallel analysis of household sanitation facilities and young children’s recent diarrhea (within two weeks preceding survey). Households that lack sanitary facilities have higher burdens of child diarrhea compared to households with a flushed toilet. We found that, compared to their peers who live in a house with a flush toilet, households using a pit/latrine or open defecation have 44 % and 27 % higher odds of having children with diarrheal disease, respectively. Although it is large and significant, the association between sanitation facilities and child diarrhea is only about one-fourth the size of the one between household sanitation facilities and NPSV, demonstrating the strength of the relationship with NPSV.

**Table 3:** Logistic regression model results of the association between household sanitary facilities and diarrhea among children under age five within the two weeks preceding the survey in India (2005-06), *N* = 25,285

	Odds ratio	
	(95 % CI)	*p*-value
Sanitation facilities		
Toilet	1	NA
Pit	1 · 44	0 · 01
	(1 · 11 – 1 · 86)	
Open air	1 · 27	0 · 004
	(1 · 08 – 1 · 49)	
*Respondent’s demographic characteristics*		
Education	1 · 00	0 · 87
	(0 · 99 – 1 · 01)	
Age	0 · 97	<0 · 001
	(0 · 96 – 0 · 98)	
Relationship status		
Never married	1	NA
Married	5 · 60	0 · 01
	(1 · 45 – 21 · 70)	
Widowed	3 · 40	0 · 12
	(0 · 73 – 15 · 79)	
Not living together	4 · 57	0 · 06
	(0 · 95 – 21 · 94)	
Caste/ religion		
Upper caste Hindu	1	NA
SC/ST Hindu	1 · 13	0 · 17
	(0 · 95 – 1 · 35)	
OBC Hindu	1 · 23	0 · 02
	(1 · 03 – 1 · 47)	
Lower class Muslim	1 · 59	<0 · 001
	(1 · 23 – 2 · 05)	
Forward class Muslim	1 · 40	0 · 003
	(1 · 12 – 1 · 76)	
Other SC/ST, Christian, Buddhist	1 · 48	0 · 02
	(1 · 06 – 2 · 05)	
Other	1 · 31	0 · 08
	(0 · 97 – 1 · 76)	
City size		
Mega city	1	NA
Large city	1 · 11	0 · 57
	(0 · 78 – 1 · 58)	
Small city	1 · 35	0 · 09
	(0 · 96 – 1 · 90)	
Large town	1 · 77	0 · 01
	(1 · 15 – 2 · 73)	
Small town	1 · 15	0 · 45
	(0 · 81 – 1 · 63)	
Rural	1 · 19	0 · 26
	(0 · 88 – 1 · 61)	
Geographic region		
North	1	NA
North-central	1 · 12	0 · 29
	(0 · 91 – 1 · 37)	
North-east	0 · 89	0 · 44
	(0 · 65 – 1 · 21)	
East	0 · 89	0 · 27
	(0 · 72 – 1 · 10)	
West	1 · 13	0 · 30
	(0 · 90 – 1 · 43)	
South	0 · 59	<0 · 001
	(0 · 47 – 0 · 74)	
*Household assets*		
Electricity in home	1 · 08	0 · 34
Roof material	(0 · 92 – 1 · 26)	
Natural	1	NA
Man-made, semi-permanent	0 · 89	0 · 31
	(0 · 72 – 1 · 11)	
Man-made, permanent	1 · 05	0 · 59
	(0 · 89 – 1 · 23)	
Constant	0 · 03	<0 · 001
	(0 · 01 – 0 · 14)	

*Note. 95 % CI in parentheses*

It is possible that Indian women who lack adequate household sanitation facilities share other traits that drive their higher risk of both living in a household without sanitary facilities and sexual violence. If this is the case, we expect there to be a comparable association between household sanitary facilities and IPSV within the prior year (Table 4). That is, a significant association between household sanitation facilities and IPSV—two factors not theoretically related—would suggest that our sanitation-NPSV results are likely due to some unmeasured characteristics or processes. We find no evidence that open defecation is significantly associated with an elevated risk of IPSV, lending support to the conclusion that open defecation places women at uniquely higher risk of one type of sexual violence: non-partner.

**Table 4:** Logistic regression model results of the association between household sanitary facilities and IPV within the 12 months preceding the survey in India (2005-06), *N* = 58,584

	Adjusted for demographic and partner characteristics, and household assets	
	Odds ratio (95 % CI)	*p*-value
Sanitation facilities		
Toilet	1	NA
Pit	0 · 79	0 · 032
	(0 · 63-0 · 98)	
Open air	1 · 00	0 · 966
	(0 · 85-1 · 18)	
*Respondent’s demographic characteristics*		
Education	0 · 96	<0 · 001
	(0 · 95-0 · 98)	
Age	0 · 96	<0 · 001
	(0 · 95-0 · 98)	
Caste/religion		
Upper Caste Hindu	1	NA
SC/ST Hindu	1 · 08	0 · 368
	(0 · 92-1 · 26)	
OBC Hindu	0 · 98	0 · 841
	(0 · 84-1 · 16)	
Lower class Muslim	0 · 74	0 · 045
	(0 · 56-0 · 99)	
Forward class Muslim	1 · 41	0 · 004
	(1 · 11-1 · 78)	
Other SC/ST, Christian, Buddhist	0 · 72	0 · 069
	(0 · 51-1 · 03)	
Other	0 · 64	0 · 010
	(0 · 45-0 · 90)	
City size		
Mega city	1	
Large city	2 · 51	<0 · 001
	(1 · 68-3 · 76)	
Small city	1 · 87	0 · 002
	(1 · 25-2 · 80)	
Large town	4 · 14	<0 · 001
	(2 · 62-6 · 55)	
Small town	1 · 96	0 · 001
	(1 · 30-2 · 96)	
Rural	2 · 20	<0 · 001
	(1 · 55-3 · 14)	
Geographic region		
North	1	
North-central	0 · 83	0 · 120
	(0 · 66-1 · 05)	
North-east	1 · 18	0 · 260
	(0 · 88-1 · 58)	
East	1 · 25	0 · 055
	(1 · 00-1 · 58)	
West	0 · 27	<0 · 001
	(0 · 20-0 · 36)	
South	0 · 31	<0 · 001
	(0 · 24-0 · 40)	
*Partner and couple characteristics*		
Education	0 · 98	<0 · 001
	(0 · 96-0 · 99)	
Age	1 · 00	0 · 854
	(0 · 99-1 · 01)	
Fertility	1 · 04	0 · 016
	(1 · 01-1 · 07)	
Husband does not live at home	1 · 07	0 · 520
	(0 · 88-1 · 29)	
*Household assets*		
Electricity in home	0 · 96	0 · 595
	(0 · 84-1 · 10)	
Roof material		
Natural	1	
Man-made, semi-permanent	0 · 96	<0 · 001
	(0 · 81-1 · 14)	
Man-made, permanent	0 · 95	0 · 448
	(0 · 82-1 · 09)	
Constant	0 · 19	<0 · 001
	(0 · 000-0 · 12)	

*Note. 95 % CI in parentheses*

## Discussion

The separate issues of household sanitation and women’s risk of sexual violence in India have received substantial attention from both scholars and policymakers in recent years [11, 16]. However, little research has asked whether the two issues are linked. Reliable statistics on NPSV are likely to be downward biased due to underreporting, particularly in the South Asian context, however, a systematic review found that 3.3 % of women in India and Bangladesh reported this type of violence – distinct from IPSV [17]. Non-profits dedicated to improving sanitation and water projects around the world have begun to include gender and violence components in their agenda [18, 19]. Though this suggests an increasing awareness of the link between NPSV and household sanitation, much more empirical research is needed on this topic. Additionally, improving a household’s sanitary conditions is not necessarily the solution to minimizing NPSV. Studies have shown that about 50 % of toilets built by Indian governmental programs are not used for their intended purpose [4, 20], and at least one study suggests that in North India, many people prefer open defecation to toilet use [21]. For toilets to protect women against NPSV, they must be used in place of open defecation. Improving the social acceptability of toilet use is thus imperative, and our study provides concrete evidence of immediate incentives for behavioral change. Our study is the first to quantify the relationship between sanitation facilities and NPSV in India and confirms the relevance of household sanitation facilities for women’s safety as well as children’s health.

Our study has some limitations. First, the data we use are from 2005-06, which unfortunately, are the most recent available data from India. Given the attention this topic has received and the fact that a new wave of data will not be available for at least another two years, we believe it is important to disseminate this information now, and that the new wave of data will only further confirm our findings. Second, we cannot assess a direct causal linkage between NPSV and toilet facilities because our data are cross-sectional; however, we establish temporal ordering by restricting our measures of NPSV in the last year among women who have resided in their current home for one year or more. Finally, we recognize that the intent of the DHS data collection was not to focus on domestic violence or sanitation, but are modules included in the survey. Thus the types of questions asked in these modules were limited in scope and depth, thus we are unable to ascertain detailed circumstances and consequences of NPSV.

## Conclusion

Our results, which suggest that women who use open defecation have twice the odds of NPSV than women who use household toilets, indicate that infrastructure improvements can provide women with some level of protection against NPSV. Our findings provide further rationale for NGOs and the Indian government to expand sanitation programs, and raise new questions about the potentially protective role of sanitation facilities in other contexts beyond India.

## Abbreviations

IPSV: Intimate partner sexual violence
MDG: Millennium development goals
NFHS: National family health survey
NPSV: Non partner sexual violence
SBM: Swachh Bharat Mission
TSC: Total sanitation campaign
